# A biometric authentication model using hand gesture images

**DOI:** 10.1186/1475-925X-12-111

**Published:** 2013-10-30

**Authors:** Simon Fong, Yan Zhuang, Iztok Fister, Iztok Fister

**Affiliations:** 1 Department of Computer and Information Science, University of Macau, Macau, SAR, China; 2 Faculty of electrical engineering and computer science, University of Maribor, Smetanova 17, 2000, Maribor, Slovenia

**Keywords:** Biometric authentication, Hand gesture, Hand sign recognition, Machine learning

## Abstract

A novel hand biometric authentication method based on measurements of the user’s stationary hand gesture of hand sign language is proposed. The measurement of hand gestures could be sequentially acquired by a low-cost video camera. There could possibly be another level of contextual information, associated with these hand signs to be used in biometric authentication. As an analogue, instead of typing a password ‘iloveu’ in text which is relatively vulnerable over a communication network, a signer can encode a biometric password using a sequence of hand signs, ‘i’ , ‘l’ , ‘o’ , ‘v’ , ‘e’ , and ‘u’. Subsequently the features from the hand gesture images are extracted which are integrally fuzzy in nature, to be recognized by a classification model for telling if this signer is who he claimed himself to be, by examining over his hand shape and the postures in doing those signs. It is believed that everybody has certain slight but unique behavioral characteristics in sign language, so are the different hand shape compositions. Simple and efficient image processing algorithms are used in hand sign recognition, including intensity profiling, color histogram and dimensionality analysis, coupled with several popular machine learning algorithms. Computer simulation is conducted for investigating the efficacy of this novel biometric authentication model which shows up to 93.75% recognition accuracy.

## Introduction

The goal of biometric authentication is the automated verification of identity of a living person by proving over some unique feature which only he possesses. One type of biometric authentication is physiological-oriented such as fingerprint, retina, iris, geometry of face, ear, hand or finger, etc. This is generally called ‘static modality’ because supposedly these biological properties do change very little or not at all over time. Moreover the biometric features are grounded from stationary body surfaces, be it an image of hand palm or the pattern of vascular veins on a hand.

**Figure 1:**
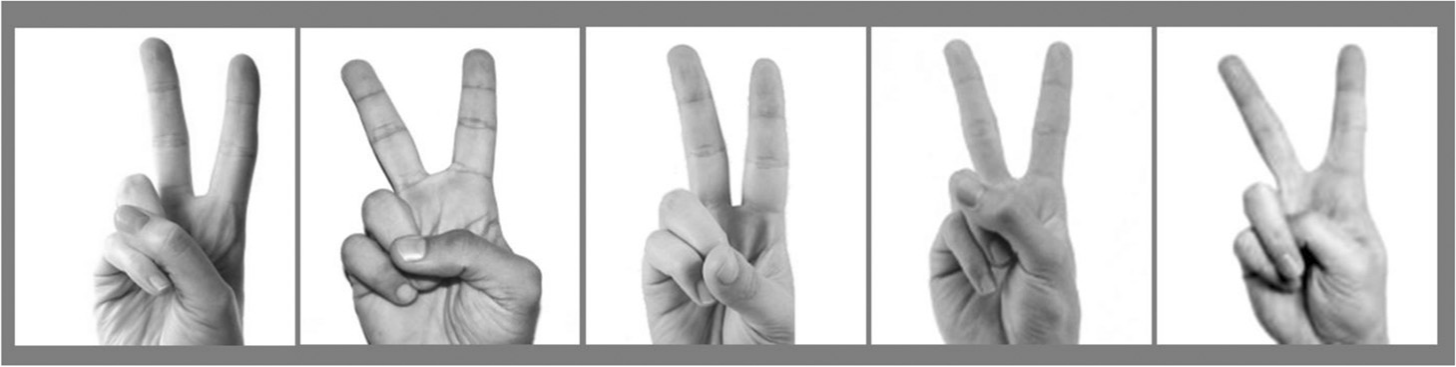
Different finger positions by different person in doing a common hand sign.

In this paper, a new concept for classifying a set of static data from hand gestures for biometric user authentication is proposed. The main novelty of this approach is in two-fold: (1) its convenience in acquiring both types of data in a single session, the allowance of certain ambiguity hence extra security in sending and testing the feature data at the classifier, and perhaps most importantly its ability to recognize the contents of the hand signs and to differentiate different signers. (2) The recognition is based on the signers’ hand shapes, or hand postures to be precise, when doing the hand signs. It is believed that by instinct everybody has his/her unique style in addition to the hand shape in performing a hand posture. For an example that is shown in Figure 1, a simple victory hand sign when made by different persons can essentially be very different in a close-up. Therefore it is supposed that the finger positions and hand postures would differ from one individual to another during communication of hand sign languages.

**Figure 2:**
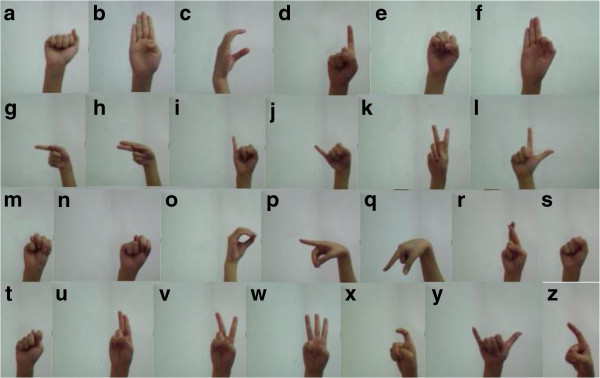
Images of the 26 letters in American Sign Language.

This new form of biometric authentication is leveraged by the prevalence of sign language. In some developed countries, signing is encouraged to learn even from the young time by toddlers [1]. Signing is not only limited to the deaf communities. In UK, schools are encouraged to explore sign language in the classroom [2], because it is an effective tool in stimulating learning of language and numeracy skills for children. In our system, the biometric model is trained to recognize 26 letters in American Sign Language (which is shown in Figure 2) by using a simple video camera to capture the real time hand gestures. However, the model provides flexibility of assigning any message to associate with any hand gesture. In other words, a user can invent his new hand gesture and associate it with a secret message in the enrollment phase of the biometric authentication. Upon testing, presenting the same gesture will display the secret message to the user provided that the gesture matches with his user ID and may be other security measures. This will be useful for two-way authentication. Alternatively, the user can be challenged to input the secret message via a keyboard (just like a password) and it will be verified together with a secret hand gesture that would be known to only the original person. In this case, it offers double security on top of the password which could be replicated or stolen.

In this paper, our study proposes a multimodal biometric approach integrating features from static hand gesture. Our proposed biometric system can be constructed as a low-cost device because it relies on an ordinary video camera in hardware and image analysis in software to extract the feature points. The image analysis is based on statistics, and therefore is relatively fast in comparison to those sophisticated image processing techniques discussed in Section 2. Hand-features biometric recognition has other advantages too when compared to other types of biometric features like face, eyes and DNA etc., in authentication. The advantages of hand-based recognition include the following: (1) Contactless capture. (2) Non-duplicability; Passwords and signatures would sometimes be needed to be printed on hard copies that could be stolen, forged and duplicated. The hand gestures however are required to be momentarily projected upon a video-camera usually at a perpendicular angle, and this process can be performed in a block box to which the hand is inserted and the actions within concealed. The image data captured would not leave behind any record in the system. The instant image would be transformed and encoded into some numeric features which are fuzzy in nature, to enable approximate-matching in the internal classifier model. In this case, the recognition system is a probabilistic model, instead of a deterministic model, as there is no need exact for matching point-to-point. In each round of hand gesture performance the encoded digest would be very slightly different; it is the underlying core patterns that would be used for authentication. This core patterns are hidden among a vast number of image features, hence they are not easily duplicated. (3) Non-repudiation; The signer/user is required on the spot to perform a hand gesture. During the authentication the presence of the signer is expected to be in person. Therefore the transaction could be proven non-repudiated with the gesture that is being authenticated can only come live from the legitimate signer. (4) Proof of liveliness; Falsification is relatively difficult on live hand gestures.

However, just as all biometric authentication approaches are far from perfect, this pioneer work on hand gesture recognition is subject to throughout studies in different aspects of security, scalability, feasibility and its related management policies. This paper however focuses on the efficacies of data pre-processing methods and the recognition of signers and the gesture contents by various popular classification algorithms. In particular, as a technical contribution by this paper, we evaluate the performance of different classifiers pertaining to the proposed hand gesture biometric authentication model.

The rest of this paper is organized as follows: in Section 2 Related Work, we discuss some related technologies for hand biometric recognition system. Our proposed bimodal hand gesture authentication system using static hand movements is presented in Section 3. Section 4 covers the computer simulation experiment implemented based on the proposed model, followed by an analysis of the experimental results. Section 5 concludes the paper.

## Related work

Recently a number of innovative methods in biometrics and biosecurity have emerged; some of them are iris recognition even after eye surgery [3], privacy-protected biometric card with medical history embedded [4], eigenbeat features of electrocardiogram [5], voice recognition [6] and hand knuckle surface recognition [7], just to name a few. As this emerging trend continues to draw attention from researchers from biomedical and information technology research communities [8], the endeavor largely develops into two groups – one is vision-based and the other is motion-based.

The motion-based group of methods is mainly those that involve generating some behavioral traits by hands. These include for example, online handwriting by waving a hand in air that simulates writing a message [9], keystroke dynamics where the intervals and speed of typing on a keyboard are measured [10], and hand motion recognition [11]. In [11], a preliminary work was done on designing a classifier system that can recognize 10 elementary gestures. The recognition was done by decoding a motion gradient orientation image into the feature vector representative of one of the 10 gestures. Since the recognition was confined by only 10 gestures, we opt to extend them into 26 hand gestures according to American Sign Language.

Nevertheless hand gesture recognition has become a mainstream research study that crosses computer vision and machine learning disciplines. One of the latest works [12] claimed that multiple classifiers are to be used as an ensemble in order to accommodate the complex interpretation of potentially many human hand gestures. The bias-variance decompositions of error for all the compared algorithms are studied and used as a guide in choosing classifier.

Alone with this research direction on recognizing hand gesture, quite a number of researchers [12-15] focus on vision-based recognition that relies solely on the image visual information for characterizing a hand gesture. The feature representations are either extracted as statistical feature vectors [16] and Wavelet Harr values [17]. Instead embracing the full set of characteristics of a hand gesture which may incur a heavy cost of computational processing, some researchers resort to checking only a subset of features that can uniquely describe a human hand. Such streamlined methods without the need of examining the whole hand include recognizing veins pattern on the dorsal surface [18-20] and identifying the geometry [21,22] of a human hand respectively.

In regards to hand gesture recognition, static hand configuration without any movement attributes to a hand posture, with each predefined posture implies a letter as shown in Figure 2. In sign language, a hand gesture is made up of a sequence of hand postures connected by continuous motions over a brief length of time. Some researchers [17] attempted to model these two by using two levels of classifiers. They advocated that using the statistical features quantitatively is insufficient to characterize hand gestures, syntactic object description by Harr-like features are used as well. Harr-like features focus more on the information within a certain area of the image rather than each single pixel. Again this suggests that only the information that can significantly describe the features should be used. Alternatively, mechanical devices are used to sense the shape of the hand, in a type of approach called glove-based [23] hand gesture recognition. A hand glove which is mounted with sensors transmits electromagnetic signals for determining the hand posture and movements. The motions measured from hand gesture are shown to give rise to linguistic information better than static postures alone [24].

Another emerging trend as observed from the literature is the hybrid use of features that are extracted from different biometric sources. These are called multimodal recognition systems, which tap on multiple biometrics for tightening up the security levels. In particular, hand and face recognitions [25,26] are commonly used together for biometric authentication. A study in [27] advocates using a multimodal biometric authentication system that is based solely on features of a human hand. The tri-modal system takes information from Eigen-coefficients of palm, fingers between first and third phalanx, and finger tips. Their testing shows that encouraging recognition rate, as these features are indeed unique for each individual.

By reviewing a wide selection of biometric authentication from above, it is clued that a good biometric authentication system should be one that uses simple but effective features. The justification in our proposed model is that our human hand contains a wide variety of measurable characteristics that can be used by biometric systems. However, for the sake of a convenient and low cost system, where the static features can be captured from the same hand of the person, we are motivated to design a hand-based system. Simple methods are preferred over complex ones for fast processing as well.

## Our proposed model

The biometric authentication system mainly has two phases: enrollment and authentication. Any new user must first record his secret hand signs at the enrollment phase. The process is basically performing the hand signs at the user’s discreet choice in front of a camera, preferably in a concealed space – e.g. a camera covered inside a non-transparent box with sufficient space for hand movement. The captured images would be examined and segmented into a list of hand signs by some image processing algorithms in the pre-processing step. The list of hand signs which are represented by segments of picture frames would be converted into feature vector known as the composite template for further actions. A composite template is the combined digital digest of features which are extracted from the hand gesture recognition process. Optionally, the meanings of the hand signs for gesture could be arbitrarily defined by the users, possibly for two-way authentication.

The composite template that was created for the purpose of registering the user is then used for training (or updating) the classifier model. At the same time his submitted identity together may be coupled with some deciphering keys and an optional PIN are sent to and stored up in a secure database for future reference. A copy of the memories that are resulted from the classifier after being trained to recognize this new user is deposited in the secure database too. The database entry is now carrying information of the user, his security keys and a copy of the classifier (sometimes in knowledge rules) that was induced to recognize his registered hand signs.

**Figure 3:**
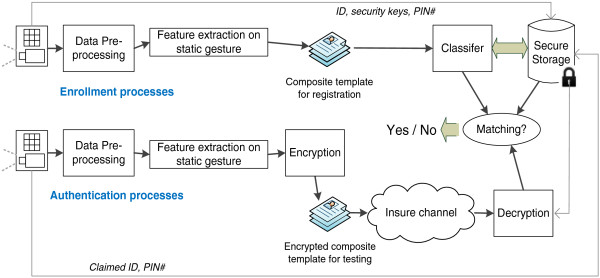
Work flow diagram that shows the enrollment processes and authentication processes of our proposed biometric authentication model.

Upon authentication challenge by the system, a user who claimed who he is, submits his claimed ID and he performs a series of hand signs in front of the camera. By referring his claimed ID to the database, the entry is retrieved if it exists. A copy of the knowledge rules that were trained to recognize his hand signs is launched to verify his hand signs under test. If the verification is successful the user who has the claimed ID is authenticated as the legitimate user, and vice-versa. The workflow is shown in Figure 3.

An optional function of the system is the encryption of the composite template, in case the authentication is an online process where the composite template needs to be transmitted across some insure communication network. The system is of a rather standard architecture that can typically be found in many biometric systems. The key components however are the data pre-processing process and the feature extraction process, which are described in details in the following section.

### Image pre-processing

For modeling hand posture (which is static hand gesture), a lightweight approach is devised in this paper. It includes image pre-processing which mainly standardizes the image of a hand, so that effective feature extraction can subsequently be applied over it. It is a lightweight approach because the image is not attempted to be reconstructed or analyzed over pixel-by-pixel. Its background is quickly removed, and the net hand image is aligned and repositioned appropriately in a standard format. Three steps are employed to pre-process hand gesture images. They are: RGB to HSV Conversion, Erosion and Dilation, and Image Alignment.

Hand gesture image is captured by a low-cost Logitech QuickCam web-camera that provides video capture with the resolution of 320×240, 15 frames-per-second. After the image capture, a HSV function is programmed to remove the background of the image. HSV helps simplifying an image by using only several from the color palette. HSV color system maps RGB values to HSV cylindrical coordinates for depicting color sensation. The color in Hue (*H*) is composed of an annular ring, which usually is represented from 0° to 360°. 0° represents red color, 120° green and 240° blue respectively. Saturation (*S*) is the concentration of the color with setting 0% means fully diluted and vise-versa. Value (*V*) is the depth of color pigment where 0% is the darkest and 100% is the brightest. The logic of the conversion is referred to [28].

$$H^{\prime }=\mathrm{Co}s^{-1}\left\{\frac{\left(R-G\right)+\left(R-B\right)}{2\sqrt{\left(R-B\right)\left(G-B\right)+{\left(R-G\right)}^{2}}}\right\}$$

$$\left\{\begin{array}{cc} H=H^{\prime } & \mathrm{if}\;B\leq G\\ H=360^{o}-H^{\prime } & \mathrm{if}\;B>G \end{array}\right)$$

$$S=\frac{\mathrm{Max}\left(R,G,B\right)-\mathrm{Min}\left(R,G,B\right)}{\mathrm{Max}\left(R,G,B\right)}$$

$$V=\frac{\mathrm{Max}\left(R,G,B\right)}{255}$$

After the HSV conversion, the image is subject to erosion and dilation with the purpose of removing noise and eliminating the background from the foreground object. For erosion, it is defined as follow. Consider in the space of two sets, *A* and *B*. When the set *B* erodes on set *A*, it can be expressed as *A*⊕*B*. *A* is the input image and *B* is the structural element when the input pixel and its surrounding pixels with respect to the structure elements 1 of the pixel values are 255, the input pixel value is set to 255. Erosion can effectively remove unnecessary elements by selecting the appropriate structural elements. Dilation is the next step after erosion. Dilation works as this: consider the two sets *A* and *B* again. *A* is the input image and *B* is the structural element when the input pixel and its surrounding pixels with respect to the pixel value of the structural elements 1 to 255 are more than one, the input value of the pixel is set to 255. It makes the image to visually expand. The aim of dilation is to fill the gaps by using the appropriate structural elements and to remove the background.

### Feature extraction

The simultaneously captured hand gesture image is passed through three stages, preprocessing, feature extraction, and finally classification. As described earlier in preprocessing stage some operations are applied to extract the hand gesture from its background and prepare the hand gesture image for the feature extraction stage. Features are extracted from several image analysis functions which are applied over two different types of image data. The first is the original intensity images of the hand, and the other type is the hand contour. While the intensity of the image map provide rich information about the shades and texture of the hand skin, the hand contour informs almost explicitly about the outlined shape of the hand.

**Figure 4:**
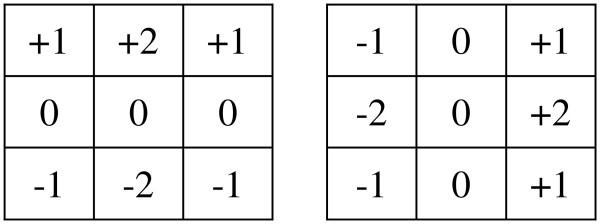
Pseudo code of the horizontal and vertical projection method.

For extracting the contour of a hand image, an effective and simple edge detection algorithm called Sobel filter is used. The filter is also known as Prewitt gradient edge detector. The filter detects and highlights the edges of an image by measuring its 2D spatial gradient according to the high spatial frequency of the nearby regions of the edges. The operation is done by a couple of 3-times-3 convolution kernels which try to find the approximate absolute gradient magnitude at each point and the orientation of that gradient. The kernels are shown in Figure 4. The gradient magnitude is thereby: $\left|g\right|=\sqrt{{\left(g^{\emptyset }\right)}^{2}+{\left(g^{+90}\right)}^{2}}$. In order to cope with the fast computation from the results of the two kernels, the gradient magnitude is approximated by using |*g*| = |*g*^∅^| + |*g*^+ 90^|. These kernels are designed to respond maximally to edges running vertically and horizontally relative to the pixel grid, one kernel for each of the two perpendicular orientations. The kernels can be applied separately to the input image, to produce separate measurements of the gradient component in each orientation (one perpendicular to the other). One kernel is simply the other rotated by 90 degrees.

**Figure 5:**
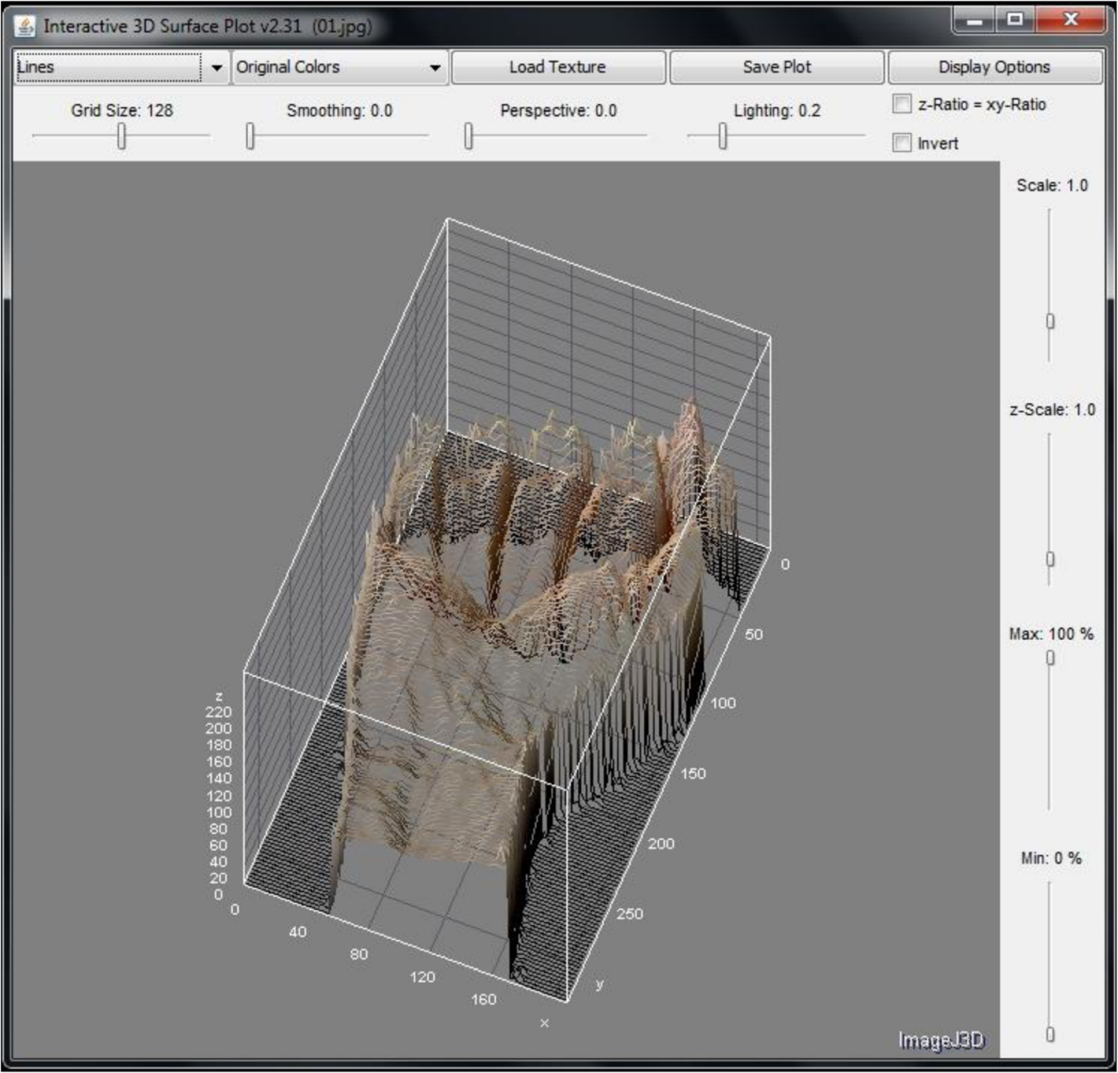
3D intensity map of hand sign of letter ‘a’ presented at +45 degrees.

**Figure 6:**
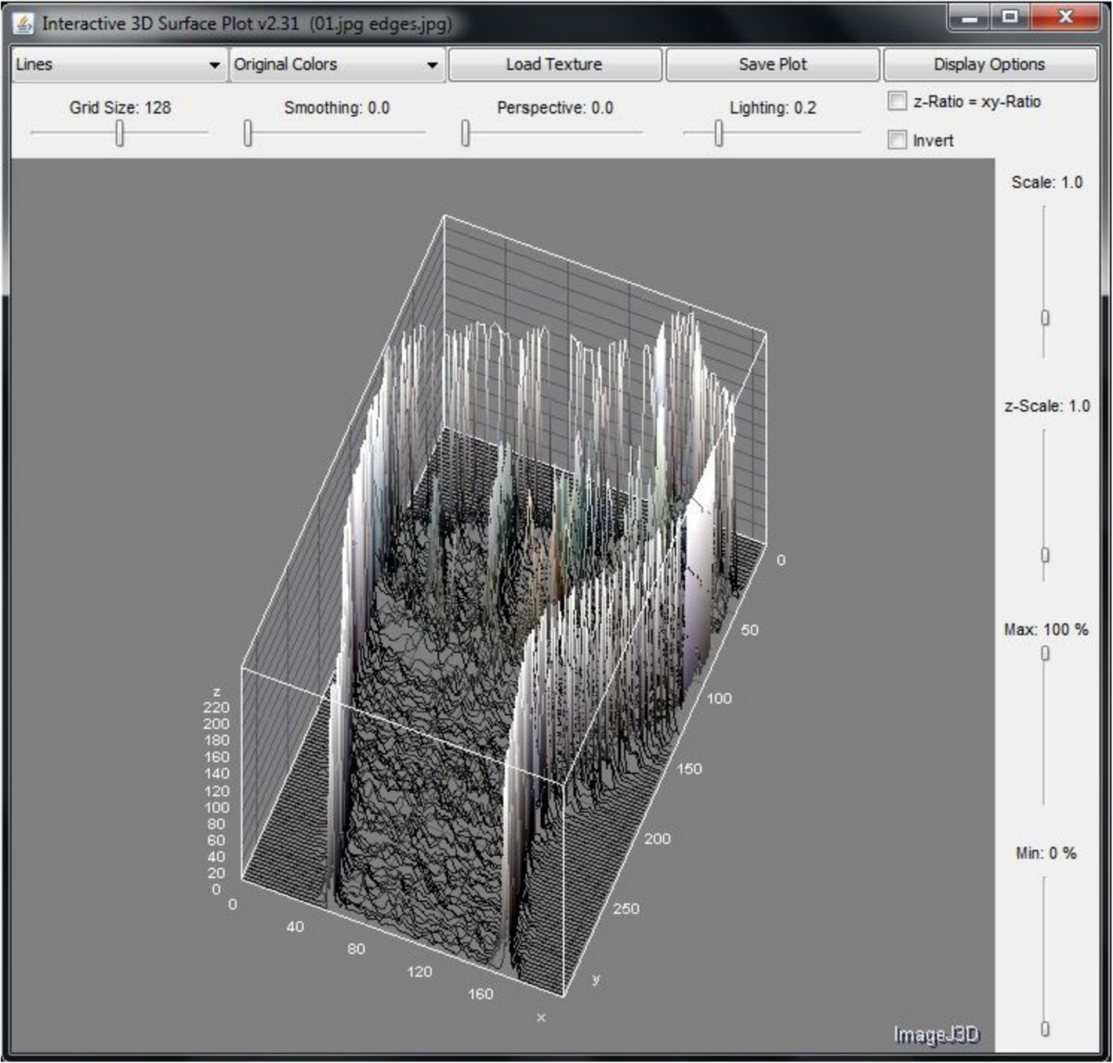
3D intensity map of hand sign of letter ‘a’ presented at +45 degrees with Sobel filter applied.

Natural edges in images often lead to lines in the output image that are several pixels wide due to the smoothing effect of the Sobel operator. However, this phenomenon which may be undesired in other applications has an advantage on amplifying the outline of a hand gesture; therefore it would be easier for a classifier to accurately differentiate different hand signs apart by recognizing their exaggerated outlines. After the hand gesture images have been processed by Sobel filter, features that describe the hand gesture are subsequently extracted. There are several types of vision-based information available via image processing algorithms which are narrated as follow, for harvest of descriptive features. They are intensity histogram and its averaged profile, color histogram, and dimensionality measures. The simplest type of vision-based information is intensity. Taken account of the intensity value 0 to 255 of each pixel across a hand gesture image, and projecting these intensity values over a 3 dimensional plot (with *x*-axis and *y*-axis being the coordinates for the 2D spatial positions of the pixels, and *z*-axis for their corresponding intensities), a visual hand gesture could well be recreated and become recognizable. The same informative information would be used for training a classifier that automatically distinguishes the gestures. Figure 5 shows a sample of intensity map for hand gesture of letter ‘a’. It can be seen clearly how the intensities mimic the detailed brightness and contrast of the surface of the hand gesture. Its counterpart after Sobel filter applied is shown in Figure 6 as well.

**Figure 7:**
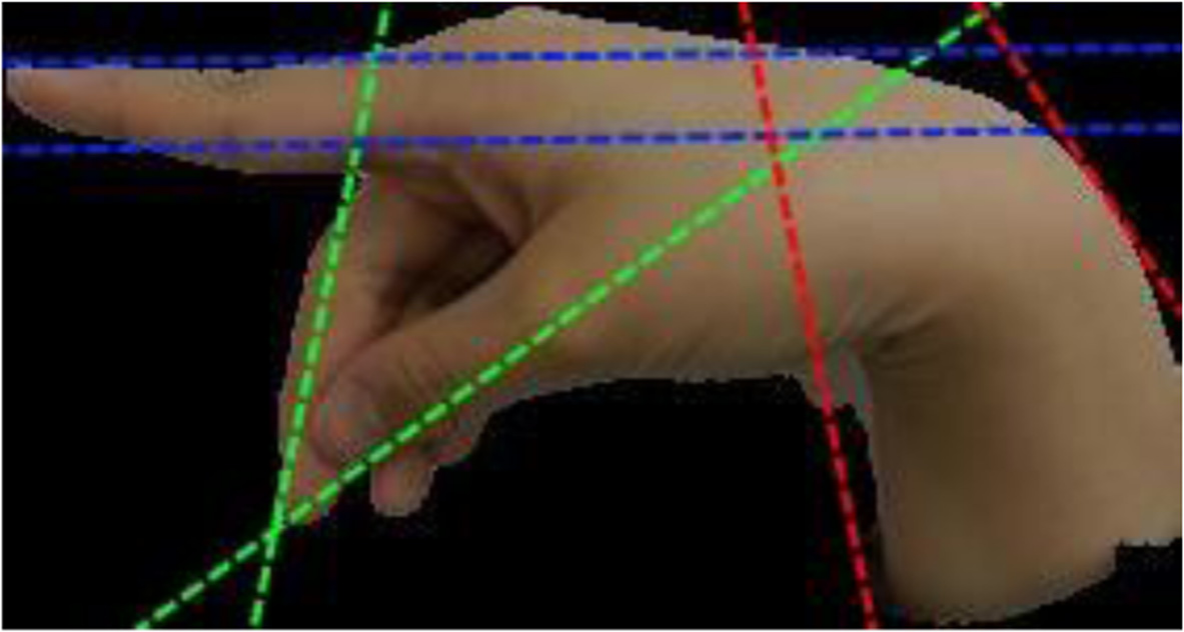
Hand gesture image of letter ‘p’ that has the directional lines added for illustration.

**Figure 8:**
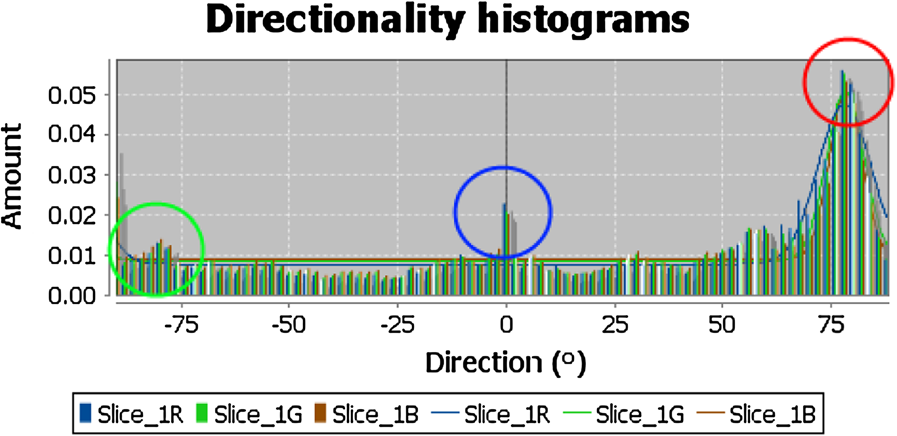
The respective Directionality Histogram of the hand gesture image of letter ‘p’.

Directionality analysis is an image processing method that can quantitatively computes a histogram of directional structures of an image. It is developed by Jean-Yves Tinevez, Max-Planck Institute of Cell Biology and Genetics, Dresden (http://fiji.sc/JeanYvesTinevez). The analysis is designed to infer the visual orientation of structures in an image. The output histogram indicates the amount of structures that are oriented across all different directions along the *x*-axis. The normalized amount of pixels of the image areas that are slanted hereto each corresponding direction, lies on the *y*-axis. Images with completely isotropic content (e.g. photo of a clear blue sky or a pile of random pebbles) are expected to produce a flat histogram. Images that contain subjects that are inclined towards some directional orientation are expected to show a histogram with some peaks at that orientation. For example, as shown in Figure 7, in the image of a hand gesture of letter ‘p’, the fingers and arm wriest are oriented mainly in three populations of pixels - the index finger is pointing almost flat about horizontally, the thumb and the middle fingers are bending towards the direction of approximately 120° assuming that the starting point is zero degree at the East direction and it goes clockwise, finally the wrist is slanted at around 75° supporting the hand. So these groups of directional oriented pixels give rise to the peaks that are shown in Figure 8, known as the directionality histogram.

The directionality analysis is implemented based on Fourier spectrum analysis. For a square image, structures with a preferred orientation generate a periodic pattern at +90° orientation in the Fourier transform of the image, compared to the direction of the objects in the input image. This plugin chops the image into square pieces, and computes their Fourier power spectra. The latter are analyzed in polar coordinates, and the power is measured for each angle using the spatial filters proposed in [29].

In addition to the histogram, the directionality analysis generates statistics pertaining to the highest peak found in the histogram as well. In the above example, the peak which has the highest peak is the arm wrist that bends at 75° because the wrist area has most pixels oriented to a common direction. The statistics generated are harvested as informative features as well as those of the other peaks, to be used for training a hand gesture classifier. The maximum peak is fitted by a Gaussian function, taking into account the periodic nature of the histogram. The ‘Direction (°)’ column reports the center of the Gaussian. The ‘Dispersion (°)’ column reports the standard deviation of the Gaussian. The ‘Amount’ column is the sum of the histogram from center-standard deviation to center+standard deviation, divided by the total sum of the histogram. The real histogram values are used for the summation, not the Gaussian fit. The ‘Goodness’ column reports the goodness of the fit; 1 is good, 0 is bad.

## Experiment

The design of the proposed biometric authentication system addresses two unique functions that (1) enable hand sign recognition via static images of hand gestures; (2) allow personal identification by distinguishing their subtle but unique behavioral patterns in posing the hand signs. During the test of static hand posture, the characteristics of a particular hand in terms of shape, intensity and color distributions of the hand, and its directional orientation in posing a hand sign are used to identify the users. Signers who are the users of the biometric authentication system pose their hand according to their enrolled secret patterns in front of a camera, to be authenticated.

Experimental evaluation is carried out as two computer performance tests: (i) predicting the identity of a signer by hand gesture; and (ii) predicting hand sign content by gesture. The data sources from which the features of the hand gestures are extracted and tested in the experiments are introduced in Section 4.1. The performance evaluation criteria are described in Section 4.2, the experimental results are reported and discussed in Section 4.3.

### Experimental data

For acquiring the static hand gesture image data, four student volunteers took turn, each to generate four sets of hand gestures for the 26 letters according to the standard American Sign Language. For each of the same letter, each student tried posing at four slightly different angles in order to enact the effect of inexactness in sign language. Then each student repeated in posing at slightly different angles.

**Figure 9:**
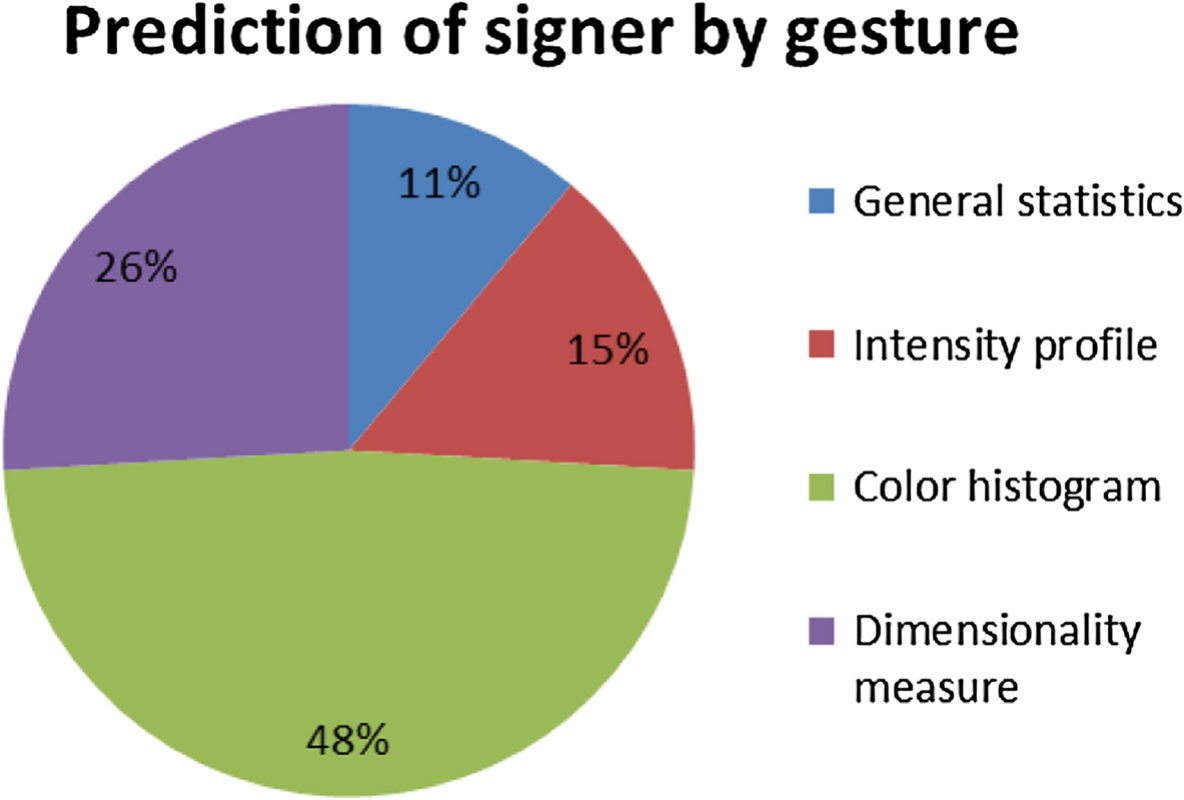
Proportion of features selected from different image analysis for classifying individual signers.

**Figure 10:**
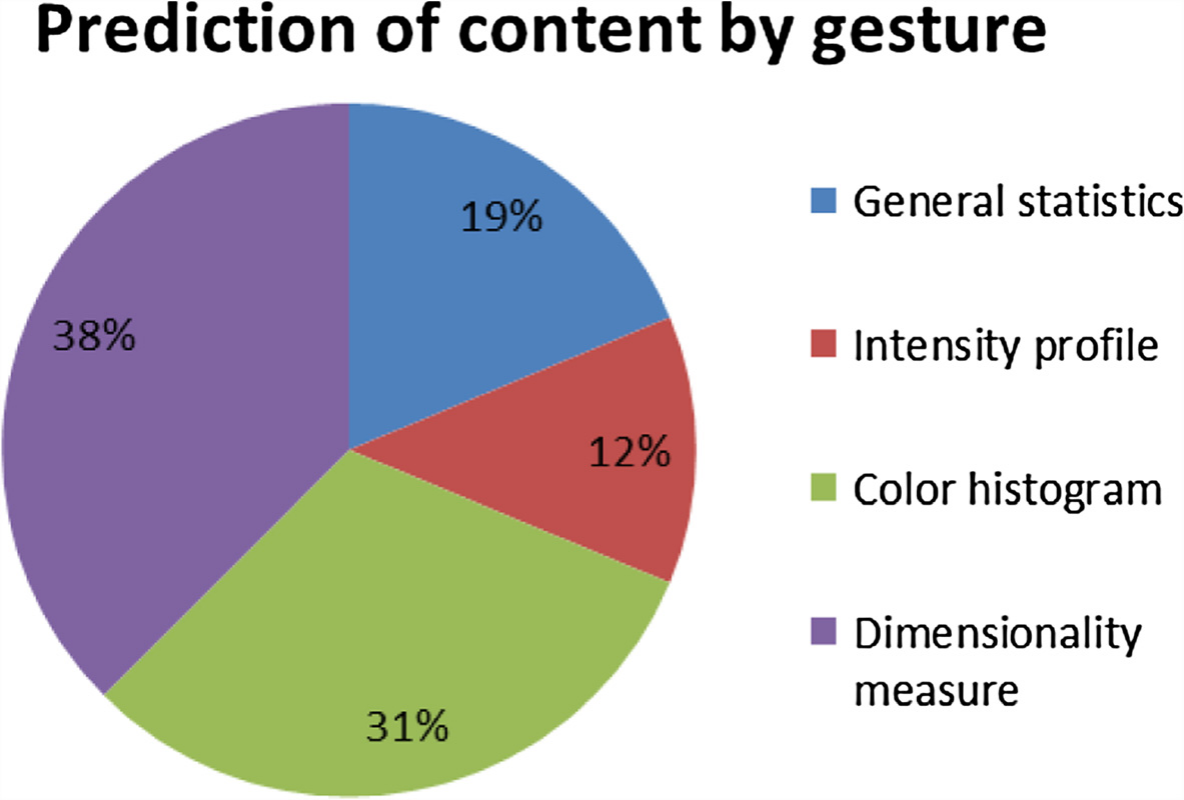
Proportion of features selected from different image analysis for classifying hand gesture contents.

In this set of data which are subject to training and testing the classifier methods, the hand contour is extracted as a feature which was treated by scaling and removal of background in real time. After that the fitted images of the gestures are processed with further feature extraction as discussed in Section 3.2. There are a total of 1,536 features that are taken from all these image analysis techniques. Dimensionality reduction is applied to remove the redundant features from training an effective classifier. The algorithm used is called Correlation based Feature Selection. The algorithm evaluates subsets of features on the basis of the following hypothesis: “Good feature subsets contain features highly correlated with the classification, yet uncorrelated to each other” [30]. The significant features are retained and used for training the classifier. Twenty-seven significant features are selected for classifier responsible for prediction of signer by gesture, and sixteen useful features are retained for prediction of content by gesture. More features are needed for predicting signer than for predicting content; it shows that it may be easier to generalize a satisfactorily accurate classifier for contents (which are limited to the distinctive shapes of 26 alphabets) than for identifying hands of each individual. The differences of each signer’s hand may be subtle and hence require more features to accomplish the training. The portions, however, by which the features are selected from different type of image analysis are shown in pie-charts in Figure 9 and Figure 10. It can be easily observed that for prediction of contents, Directionality analysis is more imperative because hand signs are distinctively different by the shapes of hand gestures. Contrariwise, significant features from color histogram dominate the feature space (by 48%) for classifying each individual’s hand, largely could be due to the different skin complexion colors.

### Performance criteria

The experiments concern about checking the performance of our proposed biometric authentication model especially the classifier. The classifier serves as a brain in predicting the identity of the signers and the content of the hand signs. Therefore its accuracy is up-most important. There are many classification algorithms available, some of which may be more suitable for hand sign pattern recognition than the others. Likewise there are multiple performance criteria by which the performance of these classifiers would be well assessed.

The performance criteria adopted here include the accuracy of the classifiers, its counterpart Mean Absolute Error, Kappa statistics, F-measure, ROC, that are being observed during the hand sign recognition. All the values of the performance results except accuracy which is in percentage, are normalized to [0,1] where 0 is the minimum and 1 is the maximum. The accuracy is simply the percentage of the correctly classified cases over the total number of testing cases. It serves as the main performance of the model indicating how ‘useful’ it is with respect to prediction. In the experiment, the option for training/testing is set to 10-fold cross validation, which is a common way in statistics to validate how well the results of a data mining model will generalize to any independent dataset. It works by randomly partitioning the full dataset to two subsets, one being the training segment and the other one being the testing segment. The testing segment serves as unseen samples for assessing the performance of the induced model; of course the testing segment has already had the predefined class labels, so the software would be able to score the accuracy of the model that was trained by the training subset. This process is repeated ten rounds, again randomly on different positions of the full dataset, in order to obtain unbiased performance results. Each time the cross-validation is performed over different random partitions. The final performance scores are those averaged over the ten rounds.

Kappa statistics is generally used in data mining, statistical analysis and even assessment of medical diagnostic tests, as an indicator on how ‘reliable’ a trained model is. It basically reflects how consistent the evaluation results obtained from multiple inter-observers are and how well they are agreed upon. A full description of the Kappa statistics can be found in [31]. Generally a Kappa of 0 indicates agreement is equivalent to chance, where as a Kappa of 1 means perfect agreement. It loosely defines here as reliability by implying a model that has a high Kappa value is a consistent model that would expect about the same level of performance (in this case, accuracy) even when it is tested with datasets from other sources. The Kappa statistics is computed here from the 10-fold cross-validation with each fold of different combination of partitions (training and testing) as different inter-observers.

In pattern recognition such as hand sign recognition in biometric authentication, precision rate or just Precision is the fraction of relevantly recognized instances. In our biometric authentication model, Precision is a measure of the accuracy provided that a specific class has been predicted. It is calculated by this simple formula: $\mathrm{Precision}=\frac{\mathrm{True}\;\mathrm{Positive}}{\mathrm{True}\;\mathrm{Positive}+\mathrm{False}\;\mathrm{Positive}}$.

Recall is defined as the fraction of relevantly retrieved instances. We can infer that the same part of both precision and recall is relevance, based on which they all make a measurement. Usually, precision and recall scores are not discussed in isolation and the relationship between them is inverse, indicating that one increases and the other decreases. Recall is defined as: $\mathrm{Recall}=\frac{\mathrm{True}\;\mathrm{Positive}}{\mathrm{True}\;\mathrm{Positive}+\mathrm{False}\;\mathrm{Negative}}$.

In a classification task, recall is a criterion of the classification ability of a prediction model to select labeled instances from training and testing datasets. A precision with score 1.0 means that every instance with label belonging to the specific class (predicted by the classifier) does indeed belong to that class in fact. Whereas a recall of score 1.0 means that each instance from that particular class is labeled to this class and all are predicted correctly, none shall be left out.

F-measure is the harmonic mean of precision and recall, that is: $F\;\mathrm{measure}=\frac{2}{\frac{1}{\mathrm{Precision}}+\frac{1}{\mathrm{Recall}}}=\frac{2\;\mathrm{Precision}\;\mathrm{Recall}}{\mathrm{Precision}+\mathrm{Recall}}$. It is also known as balanced F score or F-measure in tradition, because recall and precision are equally weighted. The general formula for *F*_*β*_ measure is: $F_{\beta }=\frac{1+\beta^{2}}{\frac{1}{\mathrm{Precision}}+\frac{\beta^{2}}{\mathrm{Recall}}}=\frac{\left(1+\beta^{2}\right)\;\mathrm{Precision}\;\mathrm{Recall}}{\beta^{2}\;\mathrm{Precision}+\mathrm{Recall}}$. As mentioned before, precision and recall scores should be taken into account simultaneously because they have a strong inter-relation essentially. Consequentially, both are combined into a single measure, which is F-measure, which is perceived as a well-rounded performance evaluation, more highly valued than the simple accuracy.

ROC is an acronym for Receiver Operating Characteristic; it is an important means to evaluate the performance of a classifier system. It is created by plotting the fraction of true positives out of the positives in *x*-axis (known as sensitivity) and the fraction of false positives out of the negatives (known as specificity) in *y*-axis. So when plotting sensitivity and specificity on a ROC plot, the curve should be the higher the better in these two directions. Theoretically any classifier will display certain trade-off between these two measures. For example, in biometric authentication system in which the user is being tested for extra precaution for security requirement, the classifier may be set to consider on more biometric features in addition to the standard ones, even though they are minor ones (low specificity) and perhaps higher influential factors are adjusted for these event variables that may directly or indirectly trigger the security alert (high sensitivity). In this paper, we used the area under the curve (AUC) as a quantitative measure to represent the probability that a classifier will rank a randomly chosen positive instance higher than a randomly chosen negative, for classifier model comparison. In general, the area under the ROC curve (AUC) is widely recognized as the measure of a diagnostic test’s discriminatory power; which in our case, the stronger the better in discriminating signers’ hand signs and their subtle behavioral patterns apart.

### Experimental results

In the experiment, ten popular machine learning algorithms are used to test four different types of prediction. The ten algorithms are chosen from the major types of classification, including decision trees, rule-based methods, kernel functions, and Bayes methods. For the details of the algorithms, readers are referred to [32] where a similar framework of experiments using classification algorithms is described. For fairness of the comparison, all the selected algorithms have been fine-tuned in advance with the best-performing parameters. The experiment for the comparison is executed in the same computing environment, including both the hardware and software; the same hand sign data as described in Section 4.1 are used for all, and the same 10 fold cross-validation option is selected across each experiment trial for each algorithm. The performance results that have been rigorously experimented over the classifiers are shown in terms of various performance criteria, in Tables 1 and 2. The results from the two Tables are done by the two different types of authentication – by the identity of the signer, and by the contents of the hand signs.

**Table 1:** Classification results of predicting signers’ identities by static hand gesture images

**Signer prediction by static gesture**										
**Group**	**Algorithm**	**Accuracy %**	**Kappa**	**Mean-abs-error**	**TP Rate**	**FP Rate**	**Precision**	**Recall**	**F-Measure**	**ROC Area**
Decision Tree	J48	78.125	0.5625	0.2316	0.781	0.219	0.791	0.781	0.779	0.725
	NBTree	90.625	0.8125	0.1216	0.906	0.094	0.908	0.906	0.906	0.973
	RandomForest	87.5	0.75	0.2138	0.875	0.125	0.881	0.875	0.875	0.941
Rule-based	DecisionTable	71.875	0.4375	0.327	0.719	0.281	0.72	0.719	0.718	0.775
	NNge	84.375	0.6875	0.1563	0.844	0.156	0.856	0.844	0.842	0.844
	Association Rules	68.75	0.375	0.3125	0.688	0.313	0.75	0.688	0.667	0.688
Functions	Perceptron	93.75	0.875	0.086	0.938	0.063	0.944	0.938	0.937	0.98
	SVM	87.5	0.75	0.125	0.875	0.125	0.881	0.875	0.875	0.875
Bayes	BayesNet	87.5	0.75	0.1177	0.875	0.125	0.875	0.875	0.875	0.977
	NaiveBayes	87.5	0.75	0.1382	0.875	0.125	0.881	0.875	0.875	0.931

**Table 2:** Classification results of predicting hand gesture contents by static hand gesture images

**Hand sign content prediction by static gesture**										
**Group**	**Algorithm**	**Accuracy %**	**Kappa**	**Mean-abs-error**	**TP Rate**	**FP Rate**	**Precision**	**Recall**	**F-Measure**	**ROC Area**
Decision Tree	J48	56.25	0.4167	0.2221	0.563	0.146	0.576	0.563	0.566	0.752
	NBTree	93.75	0.9167	0.0345	0.938	0.021	0.938	0.938	0.938	0.995
	RandomForest	78.125	0.7083	0.1953	0.781	0.073	0.776	0.781	0.777	0.942
Rule-based	DecisionTable	53.125	0.375	0.2898	0.531	0.156	0.499	0.531	0.49	0.824
	NNge	84.375	0.7917	0.0781	0.844	0.052	0.846	0.844	0.838	0.896
	Association Rules	50.	0.3333	0.25	0.5	0.167	0.333	0.5	0.375	0.667
Functions	Perceptron	93.75	0.9167	0.0538	0.938	0.021	0.95	0.938	0.937	0.964
	SVM	93.75	0.9167	0.0313	0.938	0.021	0.95	0.938	0.937	0.958
Bayes	BayesNet	93.75	0.9167	0.0303	0.938	0.021	0.938	0.938	0.938	0.995
	NaiveBayes	78.125	0.7083	0.1114	0.781	0.073	0.8	0.781	0.776	0.85

In terms of accuracy and its counterpart mean absolute error, classification algorithms such as J48, Random Forest, NNge and Perceptron performed consistently well for the two types of authentication. On the other hand, algorithms like NBTree, Decision Table, Association Rules, SVM, BayesNet and NaiveBayes performed poorly with low accuracy and high error. In contrast, the mean absolute errors for BayesNet and SVM are very little, close to almost zero, for predicting alphabets by static hand sign images. It shows that these algorithms can generalize the pattern recognition models very well for hand sign alphabets. However some algorithms which are very linear for example neural network (perceptron) and NNge that can map the decision rules by nearest neighbor methods to hyper-rectangles, can generally perform relatively well both types of prediction.

The performances by the criteria of F-measure and ROC AUC follow about the same patterns as in accuracy. For Kappa statistic, again the performance comparison follows closely the patterns of Accuracy, F-measure and ROC AUC. However, the Kappa values for the classifiers that are induced by the following three algorithms shrink very sharply, NBTree, Association Rules and BayesNet. They fail to generalize the model for a wide variety of datasets. When it comes to biometric authentication system, these algorithms should be avoided because of the poor reliability.

Largely, the two types of predictions follow a general trend; it appears that the prediction of signer by static hand gesture has a higher overall classification performance, than the prediction of alphabets by static hand sign. The Kappa value however shows that identifying contents by gesture is more reliable than identifying signers in the system. That means overall, it is more difficult for the system to recognize a human person’s behavioral pattern in hand gesture than to recognize the content of the gesture. This is further assured by the ROC AUC performance; predicting contents is always easier than predicting signers.

Interestingly a Type-I error exists in the comparison, the FP (false positive) rate is relatively the lowest in the prediction of content by gesture. The FP rate for prediction of signer by gesture, in contrast, has a higher accuracy and other performance factors than prediction of content by gesture; but prediction of signer by gesture has a higher (almost double) FP rate than prediction of content by gesture. Having a false positive rate that is higher in predicting signer by gesture, means the false alarm rate is high. A false positive occurs when the authentication system mistakenly flags a legitimate user as a wrong user. This may seem harmless when compared to the otherwise, but false positives can be a nuisance in denying access to eligible users.

## Conclusion

Biometrics is a scientific approach that involves recognizing people by measuring their physical and/or behavioral characteristics. In this paper, we proposed a novel biometric discipline that uses hand sign gestures as captured in static images in signing. The motivation of using hand sign as biometric authentication is its ease-of-use and the intrinsic behavioral characteristics in signing. Furthermore, signing can convey some secret message which tops up another level of secrecy in authentication from the underlying hand patterns and hand movements. The full potential of using hand sign biometric is yet to be unleashed, in a spectrum of security applications.

This paper serves as a preliminary research in investigating the possibilities of using hand signs as biometric authentication. Specifically we rigorously tested out two types of prediction from the perspective of an authentication system, over static hand gesture data, as well as using ten popular machine learning algorithms. The two types of prediction are: (1) identifying signer using static hand gesture, and (2) recognizing the content of the hand gesture using its static image. We argued in the paper that low operational cost is emphasized in the proposed model as it relies on only a simple video camera without expensive scanning hardware. The image processing is designed lightweight too. Simple histogram methods and directionality analysis are used in lieu of complex computational transforms.

In conclusion, the experiments showed that the results are promising overall with our proposed multimodal biometric authentication system. Maximum 93.75% accuracy could be achieved by artificial neural network, in predicting signers’ identities by static hand static gesture. The on par accuracy was observed in predicting contents by static hand sign images too. In general it was shown by the extensive experiments over various performance factors that recognizing signers (behavioral patterns) are far more difficult than recognizing the hand sign contents (character recognition). It is believed that plenty of research niches and opportunities exist, both at the level of technical methods and functional policies, by using hand sign data for biometric authentication. This paper contributes to a pioneer investigation of this novel approach.

## Competing interests

The authors declare that they have no competing interests.

## Authors’ contributions

SF initiated with the original concept of using hand gestures as a biometrics method. The experiments for validating the concepts via data-mining are carried out by supervision of SF and YZ. IF and IFJr offered ideas along the way and supported by enhancing the model as well as contributing to the mathematics of the analysis. SF drafted the manuscript. All authors read and approved the final manuscript.
